# Identification of *Pseudomonas fluorescens* Chemotaxis Sensory Proteins for Malate, Succinate, and Fumarate, and Their Involvement in Root Colonization

**DOI:** 10.1264/jsme2.ME14128

**Published:** 2014-12-10

**Authors:** Shota Oku, Ayaka Komatsu, Yutaka Nakashimada, Takahisa Tajima, Junichi Kato

**Affiliations:** 1 Department of Molecular Biotechnology, Graduate School of Advanced Sciences of Matter, Hiroshima University, Higashi-Hiroshima, Hiroshima 739–8530, Japan

**Keywords:** chemotaxis, *Pseudomonas fluorescens*, root colonization, methyl-accepting chemotaxis protein, plant-microbe interaction

## Abstract

*Pseudomonas fluorescens* Pf0-1 exhibited chemotactic responses to l-malate, succinate, and fumarate. We constructed a plasmid library of 37 methyl-accepting chemotaxis protein (MCP) genes of *P. fluorescens* Pf0-1. To identify a MCP for l-malate, the plasmid library was screened using the PA2652 mutant of *Pseudomonas aeruginosa* PAO1, a mutant defective in chemotaxis to l-malate. The introduction of Pfl01_0728 and Pfl01_3768 genes restored the ability of the PA2652 mutant to respond to l-malate. The Pfl01_0728 and Pfl01_3768 double mutant of *P. fluorescens* Pf0-1 showed no response to l-malate or succinate, while the Pfl01_0728 single mutant did not respond to fumarate. These results indicated that Pfl01_0728 and Pfl01_3768 were the major MCPs for l-malate and succinate, and Pfl01_0728 was also a major MCP for fumarate. The Pfl01_0728 and Pfl01_3768 double mutant unexpectedly exhibited stronger responses toward the tomato root exudate and amino acids such as proline, asparagine, methionine, and phenylalanine than those of the wild-type strain. The *ctaA*, *ctaB*, *ctaC* (genes of the major MCPs for amino acids), Pfl01_0728, and Pfl01_3768 quintuple mutant of *P. fluorescens* Pf0-1 was less competitive than the *ctaA ctaB ctaC* triple mutant in competitive root colonization, suggesting that chemotaxis to l-malate, succinate, and/or fumarate was involved in tomato root colonization by *P. fluorescens* Pf0-1.

Chemotaxis involves the movement of an organism toward chemical attractants and away from chemical repellents (2). Since many chemical attractants are growth substrates (15, 21, 22), chemotaxis is believed to assist bacterial cells in moving to areas suitable for growth. Bacterial chemotaxis can be also viewed as an important prelude to ecological interactions such as symbiosis, infection, and root colonization (7). Previous studies demonstrated that chemotaxis was involved in nodulation by *Rhizobium leguminosarum* (20), plant infection by *Ralstonia solanacearum* (38), and root colonization by *Pseudomonas fluorescens* (9, 25).

Certain strains of *P. fluorescens* belong to plant growth-promoting rhizobacteria (PGPR) (17, 24). They exert beneficial effects on plants by preventing the growth or actions of plant-pathogenic microorganisms such as *Pythium ultimum* (1, 30), *Gaeumannomyces graminis* (36, 37), and *Fusarium oxysporum* (6). Efficient root colonization by PGPR strains is assumed to be essential for the biocontrol of these root pathogens (35). The roles of motility and chemotaxis in root colonization by *P. fluorescens* have been reported previously. Barahona *et al.* demonstrated that a hyper motile mutant of *P. fluorescens* F113 was more competitive for rhizosphere colonization than the wild-type strain and exhibited improved biocontrol activity against *F. oxysporum* (5). Conversely, de Weert *et al.* reported that a general chemotaxis mutant (the *cheA* mutant) of *P. fluorescens* WCS365 colonized the tomato root tip less efficiently than the wild-type strain (9). Plant root exudates contain various organic compounds and the major components of the tomato root exudate are amino acids (glutamic acid, aspartic acid, leucine, isoleucine, and lysine as the major components [32]), organic acids (especially citric acid, malic acid, and succinic acid [13]), and sugars (glucose and xylose as the major components [18]). *P. fluorescens* strains have been shown to exhibit chemotactic responses toward plant seeds, root exudates, and their components (9, 25, 33, 35). Therefore, chemotaxis to these components may play a role in effective root colonization.

Methyl-accepting chemotaxis proteins (MCPs) are chemotaxis sensory proteins that are responsible for the detection of chemotactic ligands (10). Chemotactic ligands bind to the periplasmic domains of MCPs and their binding initiates chemotactic signaling. A genome sequence analysis of *P. fluorescens* Pf0-1 (accession number: CP000094) suggested the presence of 37 MCPs. We previously identified CtaA (Pfl01_4431), CtaB (Pfl01_0124), and CtaC (Pfl01_0354) as MCPs for amino acids in *P. fluorescens* Pf0-1 (25). Chemotaxis toward 18 naturally-occurring amino acids was found to be defective in the *ctaA ctaB ctaC* triple mutant of *P. fluorescens* Pf0-1 (designated FLD3), but it still showed decreased, but significant responses to proline and cysteine. In competitive tomato root colonization assays, FLD3 was less competitive than the wild-type strain, whereas this strain was more competitive than the *cheA* mutant of *P. fluorescens* Pf0-1, which is non-chemotactic, but motile. These findings suggested that chemotaxis to amino acids was involved in root colonization and there were still chemoattractants other than the 18 amino acids involved in root colonization by *P. fluorescens* Pf0-1.

We assumed that chemotaxis to root exudate components other than amino acids would also be involved in effective root colonization in *P. fluorescens*; however, no studies have investigated the relationship between root colonization and chemotaxis to sugars and organic acids in soil bacteria including *P. fluorescens*. Thus, we first measured chemotactic responses to various organic acids and sugars and found that *P. fluorescens* Pf0-1 showed strong responses to l-malate, succinate, and fumarate. We then identified MCPs for these dicarboxylic acids in *P. fluorescens* Pf0-1. We also assessed the involvement of chemotaxis to organic acids in tomato root colonization by competitive root colonization assays using *P. fluorescens* Pf0-1 mutant strains defective in chemotaxis to l-malate, succinate, and fumarate.

## Materials and Methods

### Bacterial strains, plasmids, and growth conditions

The bacterial strains and plasmids used in this study are listed in Table 1. *Escherichia coli* JM109 (27) and S17-1 (31) were used for plasmid construction and transconjugation, respectively. *P. fluorescens*, *Pseudomonas aeruginosa*, and *E. coli* strains were grown with shaking in 2×YT medium (27) supplemented with appropriate antibiotics. *P. aeruginosa* and *E. coli* strains were cultivated at 37°C, while *P. fluorescens* strains were grown at 28°C.

### Chemotaxis assay

The computer-assisted capillary assay method was performed as described previously (23). Cells in a 10-μL suspension were placed on a coverslip, and the assay was started by placing the coverslip upside down on the U-shaped spacer to fill the chemotaxis chamber with the cell suspension. Cells were videotaped over 1.5 min. Digital image processing was used to count the number of bacteria accumulating toward the mouth of a capillary containing a known concentration of an attractant plus 1% (w/v) agarose. The strength of the chemotactic response was determined by the number of bacterial cells per frame. The chemotaxis buffer was 10 mM HEPES (*N*-2-hydroxyethylpiperazine-*N*′-ethanesulfonic acid) buffer (pH 7.0). We selected test compounds for chemotactic responses by *P. fluorescens* Pf0-1 based on previous findings by Kamilova *et al.* (13), which included 21 organic acids and 5 sugars (Table 2).

### DNA manipulation

Standard procedures were used for plasmid DNA preparations, restriction enzyme digestions, ligations, transformations, and agarose gel electrophoresis (27). PCR was conducted using KOD Plus DNA polymerase (Toyobo, Tokyo, Japan) according to the manufacturer’s instructions. The oligonucleotides used for PCR are listed in Table S1. *P. aeruginosa* was transformed by electroporation as described previously (19). Plasmids were introduced to *P. fluorescens* strains by transconjugation using *E. coli* S17-1 (31).

### Plasmid construction and construction of deletion mutants of *P. fluorescens* Pf0-1

The Pfl01_0728 and Pfl01_3768 genes were amplified from the *P. fluorescens* Pf0-1 genome by PCR using the FLCP09f/FLCP09r and FLCP21f/FLCP21r primer sets, and then cloned into broad-host-range plasmid pUCP18 (29) to construct pFLCP09 and pFLCP21, respectively. Suicide plasmids pNMFL09 and pNMFL21 were constructed for unmarked gene deletion in *P. fluorescens* Pf0-1. PCR using the primer sets NM09Uf/NM09Ur and NM09Df/NM09Dr was conducted to amplify a 1.1-kb upstream region and 0.9-kb downstream region of Pfl01_0728 from the *P. fluorescens* Pf0-1 genome, respectively. The amplified upstream and downstream regions were digested with *Sal*I-*Bam*HI and *Xho*I-*Eco*RI, respectively, and ligated with the backbone of *Bam*HI-*Eco*RI-digested pK18*mobsacB* (28) to obtain pNMFL09. PCR using the primer sets NM21Uf/NM21Ur and NM21Df/NM21Dr was conducted to amplify a 1.5-kb upstream region and 1.2-kb downstream region of Pfl01_3768 from the *P. fluorescens* Pf0-1 genome, respectively. The amplified upstream and downstream regions were digested with *Xba*I-*Eco*RI and *Hin*dIII-*Xba*I, respectively, and ligated with the backbone of *Eco*RI-*Hin*dIII-digested pK18*mobsacB* to obtain pNMFL21. The chromosomal Pfl01_0728 and Pfl01_3768 genes were deleted by an unmarked gene deletion technique (28) using suicide plasmids pNMFL09 and pNMFL21, respectively.

### Preparation of the tomato root exudate

An exudate was prepared from the plant species tomato (*Solanum lycopersicum* cv. Oogatafukuju). Tomato seeds were sterilized by gentle shaking for 10 min in a solution of 8.75% (v/v) sodium hypochlorite supplemented with 0.1% (v/v) Tween 20. The sterilized seeds were soaked six times for 15 min in sterile deionized water. Nine sterile seeds were placed in 3 mL of SSE medium (4) in glass tubes (22 mm inner diameter, 25 mm outer diameter, 12 cm length), consisting of 5 mM KH_2_PO_4_, 4 mM CaSO_4_, 2 mM MgCl_2_, 2.5 mM NH_4_NO_3_, 0.5 mM KOH, 2.5 mM NaOH, and 0.02 mM Fe (as FeEDTA), and were allowed to grow in a climate-controlled growth chamber (NK system, Osaka, Japan) at 28°C with 16 h of daylight. Root exudates were collected after 18 d, evaporated to dryness at 45°C under a vacuum, dissolved in 1 mL of water, and sterilized by membrane filtration (0.45-μm pore size).

### Gnotobiotic root colonization assays

Twenty grams of quartz sand (0.1 to 0.3 mm) was placed into glass tubes (22 mm inner diameter, 25 mm outer diameter, 12 cm length) and compacted by gentle shaking. The open end of the tube was plugged with a silicone resin stopper. The tube was then autoclaved for 15 min at 121°C. Five mL PNS (plant nutrient solution) (11), consisting of 1.25 mM Ca(NO_3_)_2_, 1.25 mM KNO_3_, 0.5 mM MgSO_4_, 0.25 mM KH_2_PO_4_, and trace elements (in mg L^−1^): Fe (as FeEDTA), 4.6; B, 0.5; Zn, 0.05; Cu, 0.02; Mo, 0.01, was added to an autoclaved sand column. Tomato seeds (*S. lycopersicum* cv. Oogatafukuju) were sterilized as described in the “*Preparation of tomato root exudate*” section. To synchronize germination, seeds were placed on Petri dishes containing PNS solidified with 1.5% (w/v) Bacto^TM^ Agar (Becton, Dickinson and Company, New Jersey, USA) and incubated overnight in the dark at 4°C, followed by incubation at 28°C for 2 d. A germinated seed was aseptically placed at the center of a growth tube and 5 mm below the surface of quartz sand. Bacterial cells were grown for 14 h in 2x YT medium, centrifuged (3,300 × *g*, 2 min), washed three times with sterile deionized water, and adjusted to 10^7^ CFU mL^−1^ in PNS. In the competitive colonization assay, 50 μL of a 1:1 (v/v) mixture of the tested strain and the competitor was mixed and inoculated to the edge of a plant growth tube. The plant growth tubes were incubated in a climate-controlled growth chamber (28°C, 16 h daylight) to allow the plantlets to grow. After 3 d of growth in the plant growth tubes, the root systems of the tomato were mostly unbranched. The root tip (1 to 2 cm in length) was removed and shaken vigorously in the presence of adhering sand particles in 0.5 mL of sterile deionized water to remove bacteria. The bacterial suspension was diluted and 100 μL was plated on 2x YT agar plates. In the competitive colonization assay, the bacterial suspension was spread on 2x YT agar plates with and without rifampicin. The nonparametric Wilcoxon-Mann-Whitney test was used for statistical analyses (34).

## Results and Discussion

### Chemotactic responses of *P. fluorescens* Pf0-1 to organic acids and sugars

To identify chemoattractants other than the 18 amino acids involved in root colonization, we first measured the chemotactic responses of *P. fluorescens* Pf0-1 to components in tomato root exudates. *P. fluorescens* Pf0-1 showed the strongest response to l-malate among the compounds tested. Responses to l-malate were similar to those to serine and cysteine, the strongest attractants among amino acids (25). It also exhibited strong responses to succinate and fumarate, while citrate was a weak attractant. We did not detect any chemotactic responses of *P. fluorescens* Pf0-1 to the sugars examined.

### Identification of MCPs for l-malate

**Fig. 1:**
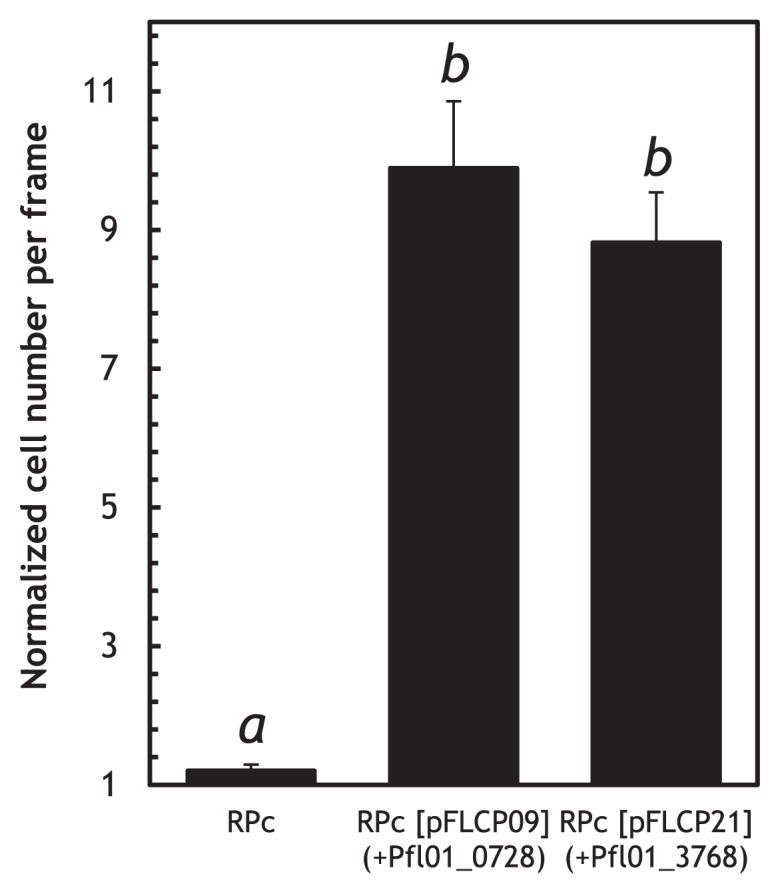
Chemotactic responses to 5 mM l-malate by *P. aeruginosa* RPc (PA2652 mutant), RPc [pFLCP09], and RPc [pFLCP21]. Digital image processing was used to count the number of bacteria around the mouth of a capillary containing 5 mM l-malate and 1% (w/v) agarose. Videotape frames were analyzed at the initiation of observations and 1 min later. Normalized cell numbers were calculated by dividing the number of bacterial cells at 1 min by that at the initiation of observations. Vertical bars represent the standard errors of measurements from two independent experiments conducted in triplicate. Different letters indicate significant differences as calculated by the Student’s *t*-test (*P* < 0.01).

**Fig. 2:**
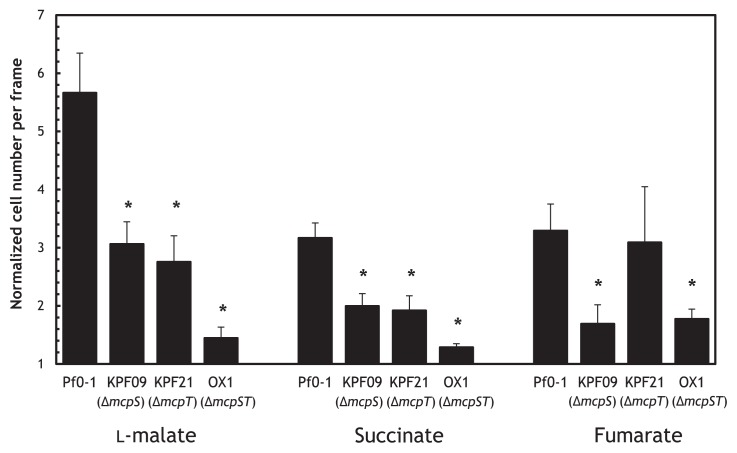
Chemotactic responses to 5 mM l-malate, 5 mM succinate, and 5 mM fumarate by *P. fluorescens* Pf0-1 wild-type, KPF09 (the *mcpS* single mutant), KPF21 (the *mcpT* single mutant), and OX1 (the *mcpS mcpT* double mutant). Videotape frames were analyzed at the initiation of observations and 1 min later. Vertical bars represent the standard errors of measurements from at least two independent experiments conducted in triplicate. Asterisks indicate the chemotactic responses in the wild-type and mutants were significantly different (Student’s *t*-test, *P* < 0.05).

To identify a MCP(s) for organic acids, we constructed a plasmid library of thirty-seven *P. fluorescens* Pf0-1 putative *mcp* genes by cloning their PCR products into pUCP18. *P. aeruginosa* PAO1 PA2652 has been identified as a MCP for malate (3). Therefore, we introduced the plasmid library to the PA2652 deletion mutant of *P. aeruginosa* PAO1 and examined the resulting recombinant strains for their chemotactic responses to l-malate. Only the introduction of the *P. fluorescens* Pf0-1 Pfl01_0728 and Pfl01_3768 genes restored the ability of the *P. aeruginosa* PAO1 PA2652 mutant to respond to l-malate (Fig. 1). To confirm the functions of Pfl01_0728 and Pfl01_3768 as MCPs for l-malate, we disrupted the chromosomal Pfl01_0728 and Pfl01_3768 genes in *P. fluorescens* Pf0-1 to construct Pfl01_0728 and Pfl01_3768 single mutants (designated KPF09 and KPF21, respectively) and tested them for chemotactic responses to l-malate. The responses of both mutants to l-malate were weaker than that of the *P. fluorescens* Pf0-1 wild-type strain (Fig. 2). Moreover, the Pfl01_0728 and Pfl01_3768 double mutant (designated OX1) did not show any responses to l-malate, confirming that Pfl01_0728 and Pfl01_3768 were the major MCPs for l-malate in *P. fluorescens* Pf0-1. Both of the single mutants showed significantly weaker responses to succinate than the wild-type strain while the double mutant exhibited no response to succinate (Fig. 2). The Pfl01_0728 single mutant (KPF09) showed markedly decreased responses to fumarate, while the deletion mutation of Pfl01_3768 did not significantly affect chemotactic responses to fumarate (Fig. 2). We demonstrated that Pfl01_0728 acted as the major MCP for succinate and fumarate as well as l-malate while Pfl01_3768 was the MCP for l-malate and succinate in *P. fluorescens* Pf0-1.

MCPs are membrane-spanning homodimers and the typical features of MCPs are as follows: a positively charged N terminus followed by a hydrophobic membrane-spanning region, a hydrophilic periplasmic domain, a second hydrophobic membrane-spanning region, and a hydrophilic cytoplasmic domain (10). Chemotactic ligands bind to the periplasmic domains of MCPs and their binding initiates chemotactic signaling. The diverse ligand specificities among MCPs reflect amino acid sequence diversities in the periplasmic domains of MCPs. Both Pfl01_0728 and Pfl01_3768 are typical MCPs and showed the features described above. We performed a BLASTP analysis on the protein database in the National Center for Biotechnology Information using the putative periplasmic domains of Pfl01_0728 (249 amino acids, residues 42 to 290 of Pfl01_0728) and Pfl01_3768 (171 amino acids, residues 33 to 203) as query sequences. The BLASTP analysis revealed that there was no significant similarity between the periplasmic domains of Pfl01_0728 and Pfl01_3768 and other *P. fluorescens* strains including F113 (accession number of the genomic sequence: CP003150), SBW25 (AM181176), WH66 (AEAZ00000000), and A506 (CP003041) possess orthologs of both Pfl01_0728 and Pfl01_3768 with up to 86% identity. The periplasmic domain of Pfl01_0728 showed 49 and 48% identities to *Pseudomonas putida* KT2440 McpS (PP_4658) and *P. putida* F1 McfS (Pput_4520), respectively, both of which were previously reported to be MCPs for malate, succinate, and fumarate (16, 26). The Pfl01_3768 periplasmic domain shares 57% identity with that of *P. aeruginosa* PAO1 PA2652, which has already been identified as a MCP for malate (3). Based on these findings, the Pfl01_0728 and Pfl01_3768 genes were designated *mcpS* and *mcpT*, respectively. All these MCPs sensed malate. *P. putida* KT2440 McpS sensed succinate and acetate, while *P. putida* F1 McfS also sensed succinate, citrate, and fumarate as well as malate. *P. aeruginosa* PAO1 did not possess an ortholog of McpS/McfS while *P. putida* KT2440 and *P. putida* F1 did not possess an ortholog of PA2562. *P. fluorescens* Pf0-1 had orthologs of both McpS/McfS and PA2562. Genomic data indicated that other *P. fluorescens* strains also had both McpS/McfS and PA2562 orthologs. These results imply that chemotaxis to organic acids plays an important role in *P. fluorescens* strains.

### The *mcpS mcpT* double mutant exhibited enhanced chemotactic responses to the tomato root exudate and specific amino acids

**Fig. 3:**
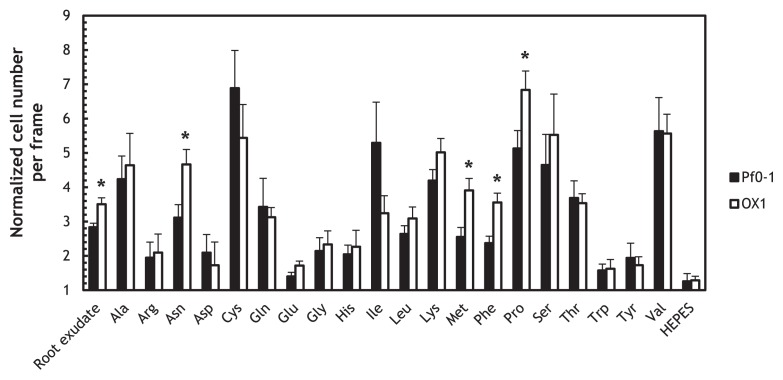
Chemotactic responses of *P. fluorescens* Pf0-1 wild-type (solid bars) and OX1 (open bars) to the root exudate, 0.5 mM naturally-occurring amino acids, and 10 mM HEPES (control). Videotape frames were analyzed at the initiation of observations and 1 min later. Vertical bars represent the standard errors of measurements from at least two independent experiments conducted in triplicate. Asterisks indicate the chemotactic responses in the wild-type and OX1 were significantly different (Student’s *t*-test, *P* < 0.05).

We previously showed that the *ctaA ctaB ctaC* triple mutant exhibited decreased chemotactic responses to the tomato root exudate (25). A previous study reported that organic acids including l-malate and succinate were components in the root exudate (13); therefore, we predicted that the *mcpS mcpT* mutant (OX1) would also show decreased responses to the tomato root exudate. However, the responses of OX1 to the tomato root exudate were significantly stronger than those of wild-type Pf0-1 (*P* < 0.05) (Fig. 3). To determine whether chemotactic responses were generally enhanced in the *mcpS mcpT* mutant, we measured the chemotactic responses of OX1 to 20 naturally-occurring amino acids (Fig. 3). The results obtained indicated that OX1 only showed significantly stronger responses to specific amino acids, including proline, phenylalanine, methionine, and asparagine, that those of wild-type Pf0-1 (*P* < 0.05). We previously demonstrated that CtaA, CtaB, and CtaC sensed 16 amino acids, 16 amino acids, and 5 amino acids, respectively (25). Only CtaA sensed proline, phenylalanine, methionine, and asparagine. However, since the *mcpS mcpT* double mutation did not affect chemotactic responses to 16 amino acids, it is currently difficult to explain the enhanced responses to four amino acids in OX1 only by the upregulation of *ctaA*. We speculate that the *mcpS mcpT* mutation may have enhanced the expression of MCP(s) for amino acids other than CtaA, CtaB, and CtaC, and that it may be responsible for the increased responses observed to these four amino acids in OX1.

### Involvement of chemotaxis to malate, succinate, and/or fumarate in tomato root colonization by *P. fluorescens* Pf0-1

**Fig. 4:**
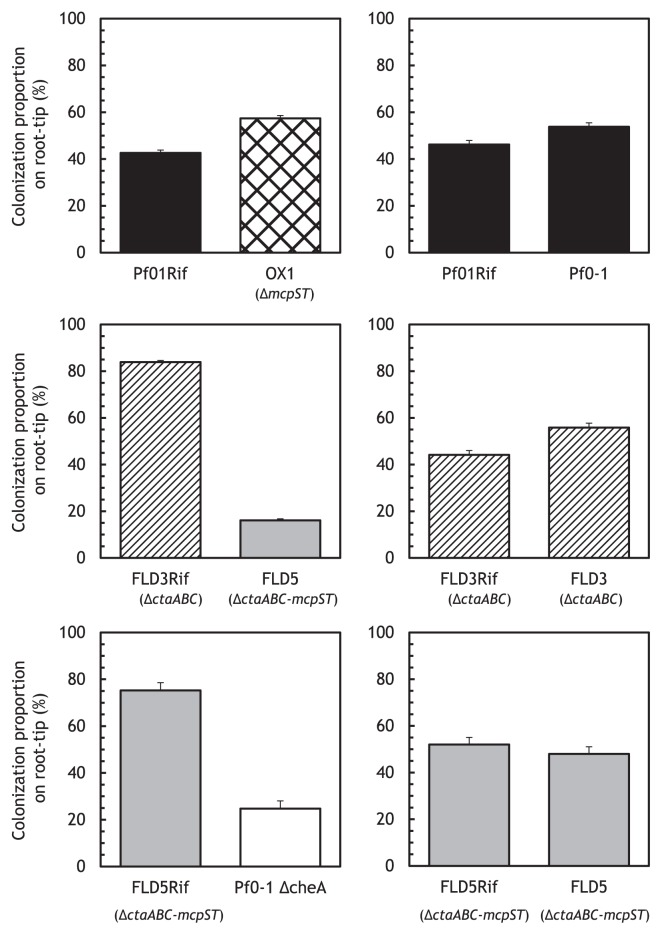
Tomato root tip colonization by *P. fluorescens* strains in competition with Rif^r^ spontaneous mutants. Root systems were sampled in at least three independent experiments conducted in triplicate. Vertical bars represent the standard errors of measurements. Significant differences were observed in colonization between Pf01Rif and OX1 (*P* < 0.05), FLD3Rif and FLD5 (*P* < 0.01), and FLD5Rif and Pf0-1 Δ*cheA* (*P* < 0.01). The nonparametric Wilcoxon-Mann-Whitney test was used for statistical analyses.

**Fig. 5:**
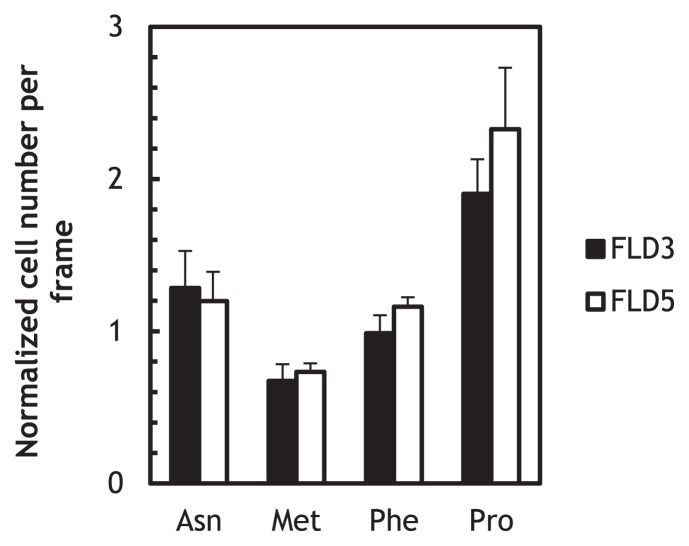
Chemotactic responses of *P. fluorescens* FLD3 (solid bars) and FLD5 (open bars) to 0.5 mM l-asparagine, 0.5 mM l-methionine, 0.5 mM l-phenylalanine, and 0.5 mM l-proline. Videotape frames were analyzed at the initiation of observations and 1 min later. Vertical bars represent the standard errors of measurements from at least two independent experiments conducted in triplicate.

To investigate whether chemotaxis to l-malate, succinate, and/or fumarate were involved in tomato root colonization, we performed competitive root colonization assays by inoculating tomato seedlings with a 1:1 mixture of a test strain and competitor strain in a gnotobiotic root colonization system. We confirmed that there were no significant differences in growth in PNS medium supplemented with glucose between mutants and the wild-type Pf0-1 (Fig. S1). Since Pf01Rif and FLD3Rif (spontaneous rifampicin-resistant mutants of Pf0-1 and FLD3 [the *ctaA ctaB ctaC* mutant of Pf0-1]) competed fully with Pf0-1 and FLD3, respectively (Fig. 4), we used Pf01Rif and FLD3Rif as competitor strains in competitive colonization assays to distinguish competitor strains from test strains. OX1 (the *mcpS mcpT* mutant) was significantly more competitive than Pf01Rif (Fig. 4). Since OX1 exhibited enhanced chemotactic responses to four amino acids, it was not possible to assess whether chemotaxis to organic acids was involved in root colonization based on this result. We found that the *mcpS mcpT* double mutation did not affect chemotactic responses to the four amino acids in the *ctaA ctaB ctaC* mutant background (*i.e.* the *ctaABC-mcpS mcpT* quintuple mutant, designated FLD5) (Fig. 5). Therefore, we evaluated the contribution of chemotaxis toward dicarboxylic acids to root colonization in competitive root colonization assays between the *ctaABC* triple mutant and *ctaABC-mcpS mcpT* quintuple mutant (FLD5) (Fig. 4). The quintuple mutant FLD5 showed a significantly reduced ability to colonize tomato root in competitive root colonization assays using the *ctaABC* triple mutant (FLD3) as the competitor strain (*P* < 0.01) (Fig. 4). This result indicated that chemotaxis to l-malate, succinate, and/or fumarate was involved in effective root colonization by *P. fluorescens* Pf0-1. However, the quintuple mutant was more competitive than the *cheA* mutant in the competitive root colonization assay, suggesting that there were chemotactic compounds other than the 18 amino acids and dicarboxylic acids (l-malate, succinate, and fumarate) that were involved in root colonization by *P. fluorescens* Pf0-1. Kamilova *et al.* reported that *F. oxysporum* altered the metabolism of organic acids in tomato plants and increased the amount of succinate (14). In such a case, chemotaxis to succinate may contribute more to the migration of *P. fluoresces* to plant roots in soil.

To identify the compounds involved in root colonization by *P. fluorescens* Pf0-1, we mainly focused on the strong chemoattractants of this bacterium in our present and previous studies (25). Strong chemoattractants were selected based on chemotaxis assay data using *P. fluorescens* Pf0-1 cells grown in basal salt medium (T_0_ medium). However, the chemotactic response profile by cells grown in T_0_ medium may not necessarily be identical to that by cells existing in the gnotobiotic root colonization assay system due to a difference in the *mcp* gene expression profile. *mcp* gene expression profiles in soil environments should be taken into consideration when identifying additional chemotactic compounds involved in root colonization by *P. fluorescens*.

## Supplementary Information



## Figures and Tables

**Table 1:** Bacterial strains and plasmids used in this study

Strain or plasmid	Relevant characteristics	Reference (s)
*Pseudomonas fluorescens*		
Pf0-1	wild-type strain	(8)
Pf01Rif	Pf0-1 derivative, spontaneous rifampicin-resistant mutant	(25)
FLD3	Pf0-1 derivative, Δ*ctaA* (Pfl01_4431) Δ*ctaB* (Pfl01_0124) Δ*ctaC* (Pfl01_0354)	(25)
FLD3Rif	FLD3 derivative, spontaneous rifampicin-resistant mutant, Δ*ctaA* Δ*ctaB* Δ*ctaC*	(25)
KPF09	Pf0-1 derivative, Δ*mcpS* (Pfl01_0728)	This study
KPF21	Pf0-1 derivative, Δ*mcpT* (Pfl01_3768)	This study
OX1	Pf0-1 derivative, Δ*mcpS* Δ*mcpT*	This study
FLD5	Pfl0-1 derivative, Δ*ctaA* Δ*ctaB* Δ*ctaC* Δ*mcpS* Δ*mcpT*	This study
FLD5Rif	FLD5 derivative, spontaneous rifampicin-resistant mutant, Δ*ctaA* Δ*ctaB* Δ*ctaC* Δ*mcpS* Δ*mcpT*	This study
Pf0-1 Δ*cheA*	Pf0-1 derivative, Δ*cheA* (Pfl01_1566)	(25)
*Pseudomonas aeruginosa*		
PAO1	wild-type strain	(12)
RPc	PAO1 derivative, PA2652 gene knockout mutant	This study
*Escherichia coli*		
JM109	*recA1*, *endA1*, *gyrA96*, *thi-1*, *hsdR17* (r_k_^−^ m_k_^+^), *e14*^−^ (*mcrA*^−^), *supE44*, *relA1*, Δ (*lac-proAB*)/F′[*traD36*, *proAB^+^*, *lacI^q^*, *lacZ* ΔM15]	(27)
S17-1	MM294, RP4-2 Tc::Mu-Km::Tn7 chromosomally integrated	(31)
Plasmids		
pUCP18	*Escherichia-Pseudomonas* shuttle vector; Cb^r^	(29)
pFLCP09	pUCP18 with a 2.5 kb PCR fragment containing *mcpS* (Pfl01_0728); Cb^r^	This study
pFLCP21	pUCP18 with a 2.1 kb PCR fragment containing *mcpT* (Pfl01_3768); Cb^r^	This study
pK18*mobsacB*	Km^r^ pUCP18 derivative, *lacZ*α, *mob* site, *sacB*	(28)
pNMFL09	pK18*mobsacB* with a 1.1-kb PCR fragment upstream of *mcpS* and 0.9-kb PCR fragment downstream of *mcpS*; Km^r^	This study
pNMFL21	pK18*mobsacB* with a 1.5-kb PCR fragment upstream of *mcpT* and 1.2-kb PCR fragment downstream of *mcpT*; Km^r^	This study
pNMPAR	pK18*mobsacB* with a 1.6-kb PCR fragment upstream of the PA2652 gene and 1.2-kb PCR fragment downstream of the PA2652 gene; Km^r^	This study

Cb^r^, carbenicillin resistance; Km^r^, kanamycin resistance.

**Table 2:** Chemotactic responses of *P. fluorescens* Pf0-1 to plant-associated compounds

Compounds^a^	Chemotactic responses^b^
l-(−)-malic acid	+ + +
d-(+)-malic acid	−
fumaric acid	+ +
maleic acid	−
succinic acid	+ +
oxaloacetic acid	+
*trans*-aconitic acid	−
citric acid	+
acetic acid	−
benzoic acid	−
*o*-hydroxy benzoic acid	−
*m*-hydroxy benzoic acid	−
*p*-hydroxy benzoic acid	−
l-pyroglutamic acid	−
phenoxyacetic acid	−
syringic acid	−
protocatechuic acid	−
d-(−)-quinic acid	−
*trans*-ferulic acid	−
shikimic acid	−
γ-aminobutyric acid	−
glucose	−
fructose	−
maltose	−
ribose	−
xylose	−
Serine	+++
Cysteine	+++

a Compounds other than *trans*-ferulic acid were used at a concentration of 5 mM. *Trans*-ferulic acid was provided as a saturated solution in 10 mM HEPES buffer (pH 7.0).

b Videotape frames were analyzed at the initiation of observations and 1 min later. Normalized cell numbers were calculated by dividing the number of bacteria at 1 min by that at the initiation of observations. The value of the normalized cell number was represented by the symbols, as follows: + + + > 4; 4 ≥ + + > 2; 2 ≥ + > 1.5; 1.5 ≥ − > 0. Serine and cysteine were positive controls.
