# Supplementary material for: Identification of Pseudomonas fluorescens Chemotaxis Sensory Proteins for Malate, Succinate, and Fumarate, and Their Involvement in Root Colonization
Source: Microbes Environ. 2014 Dec 10;29(4):413–9. doi: 10.1264/jsme2.ME14128 (PMC4262366; doi:10.1264/jsme2.ME14128)
Supplement: Supplementary file 1 [file 29_413_s1.pdf]

Table S1 Oligonucleotides used in this study

Primer name	Sequence (5’ to 3’)
For construction a plasmid library of 37 putative *mcp* genes.	
FLCP01f	ACGTAGAATTCGAATCTGCCAGAAATGCCGTCC
FLCP01r	ACGTAGGATCCGATGGGCAATGTGCTGAAGCTG
FLCP02f	ACGTACTGCAGCCGAGGTGTTTGGTCGTGATAC
FLCP02r	ACGTAGGATCCGTGGCGGTCGAAATAACAGCAG
FLCP03f	AAGTGCTGGACGTGATGAAGAAC
FLCP03r	ATGAATTCACTGATCCTCGGCCTCGAAC
FLCP04f	TAGGATCCCAAATACGCACACCACCTCAAG
FLCP04r	ATACTCGAGCGGAAAGACGGGCACAAAC
FLCP05f	ATGAATTCCGCAGCAGATTGAAATGAAGAAG
FLCP05r	TAGGATCCCGTGAGCCGTTGGAGAAAC
FLCP06f	TAGGATCCGCAGCCTGTGTACGAAGAAGTG
FLCP06r	ATAGTCGACTGATTGTGGTGTCGGGATTG
FLCP07f	TAGGATCCTTTCCGGTGGATAGCACAAAC
FLCP07r	ATGAATTCGCAATAAATTCAGGCGCTTACC
FLCP08f	ATACTGCAGGGATTGATCGTAGCCGCTTG

21	FLCP08r	TAGGATCCCAAGGAAACGGGTGAGAAATAGG
22	FLCP09f	TAGGATCCGGATTCTGGCGTAGCCGTAG
23	FLCP09r	ATGAATTCGCCAGAGGGTCAAAGGAATG
24	FLCP10f	ATTAGATCTGGTCCCGAAGGCATCAATAC
25	FLCP10r	ATACTCGAGAGCGATCCGTTCAGTTGCTC
26	FLCP11f	TAGGATCCACGCCGAACTCAACAAACC
27	FLCP11r	TATAAGCTTATGCGAAACAGCAGAAACAAC
28	FLCP12f	TAGGATCCAATCGAAACGCGGAAGAAAC
29	FLCP12r	ATGAATTCTCACCCGCCTCACAACAAG
30	FLCP13f	ATGAATTCCGGGCGATTCAACAAACTC
31	FLCP13r	TAGGATCCACACAAAGCAAGCGCATCAC
32	FLCP14f	TAGGATCCTTCTCCTCGGACTTCCTTTCTC
33	FLCP14r	ATGAATTCCATTTCGGCCTGTTCACCTATC
34	FLCP15f	ATGAATTCTAGCCACATCTCCCGCATC
35	FLCP15r	TAGGATCCACAACAACAAGGAATACCCATGAAC
36	FLCP16f	TAGGATCCGCCAGGGAGGAAATGAACAG
37	FLCP16r	ATGAATTCACATTCACGATACAAACCGACAAC
38	FLCP17f	ATACAGTTTCACCTCGAAGAACACC
39	FLCP17r	ATGAATTCCAGAGAGCGATGCACAGAGG
40	FLCP18f	ATACTGCAGTCAAAGACCCAGAGGAAATCAAG

41	FLCP18r	ATTGGATCCGATACAGAAATCAGGCGCACAC
42	FLCP19f	GTGGTTCGAGAGAGACCGATG
43	FLCP19r	ATTGAATTCAAGAACTGGCTGAAGAGATGTGG
44	FLCP20f	ATTGGATCCTGCTGACCTCCCAACTTCATTAG
45	FLCP20r	ATTGAATTCCGCATCGGTGGAAGAAGAG
46	FLCP21f	ACGTAGGATCCTCGAGAGATACGCCGACATGAG
47	FLCP21r	ACGTAGAATTCGCTGCGATGGTCCTTTAACTCC
48	FLCP22f	ATTGAATTCCGCCATAAAGAAAGCCGAAAC
49	FLCP22r	ATTGGATCCTGATTTCCGTCATTCCACAAAC
50	FLCP23f	ATTGAATTCGACCGAAGTGTTGCGTGAAG
51	FLCP23r	ATTGGATCCCCCGAGACGATCAACAGAAAC
52	FLCP24f	ATAGGTACCGGCTTTGGCTTTCACCACTC
53	FLCP24r	TATGAGCTCGCTGCGGTGTTCAGTTGTCC
54	FLCP25f	ATTGGATCCATCAACAGCGTGACCCATCC
55	FLCP25r	ATACTGCAGAGGTGAAAGCCCATCAGGAC
56	FLCP26f	TATGGATCCATCAAACCTCTTACGCCGATG
57	FLCP26r	TAACTCGAGCCGAAGGTTGTGTTGAGGTG
58	FLCP27f	ATTGGATCCCATCCTGGCTTTCCTGCTTC
59	FLCP27r	ATTGAATTCGGCCACAATTCACGTCTTACC
60	FLCP28f	TAACTCGAGGCGGATTGGTCAGCTCTTG

FLCP28r	TATGGATCCCGCGCTGTCTATGCTCATTC
FLCP29f	ATTGGATCCCCGATCTATCTGGCGATTGTG
FLCP29r	TAAGTCGACGAGTGCGGCTTGAATGGAC
FLCP30f	ACGTAGGATCCAGCTAAAGGTGACAGATGCGAC
FLCP30r	ACGTACTGCAGGCGTTGATCTCCCTTGGTTGAC
FLCP31f	ATTGGATCCCTATCCCGCCGAAATCAATC
FLCP31r	ATTGAATTCCATTCCTGCGTTCAAGTTTCC
FLCP32f	ATTGAATTCACATTCATATCCACCGACAAACC
FLCP32r	ATTGGATCCGAGACGCCACTGCATGTTC
FLCP33f	ATTGAATTCTTTAGCAGGGCACCAATCC
FLCP33r	ATTGGATCCCGGGTTCCTACAGAATCAGACC
FLCP34f	CCTGATGCACGCAAGGAAC
FLCP34r	ATAGGATTCCCTCCTTCACCTACAACATTGCCTAC
FLCP35f	TAACTCGAGCAACCGAAGGAGAATGCAATG
FLCP35r	ATTAGATCTGGGTCGTTAGCTCAGTTGGTAGAG
FLCP36f	ATTGGATCCGATTTCACCCTTGACCCATTC
FLCP36r	ATTGAATTCGGCGGGTATTGGTGGTAGAG
FLCP37f	ATTGGATCCAAGATGCACATGACCAACACC
FLCP37r	ATTGAATTCATCGCTCAAGTATTCGGTTTCAC

For construction of unmarked gene deletion mutants

NM09Uf	TAAGTCGACGCCGCATCGAATGACAAG
NM09Ur	TATGGATCCCGAAAGCCACCACCACAAAC
NM09Df	TATGAATTCATGGCGCAGGTATTTGAGCAG
NM09Dr	TAACTCGAGCGCAGCCTCATCAGAGGTAAG
NM21Uf	ACGTATCTAGAAACGATCACGGGAGTTAAAGGACC
NM21Ur	ACGTAGAATTCTTGTGGCGGATATGCCTTGTTCAG
NM21Df	ACGTAAAGCTTGGCACTCACTTCATCTAACGAC
NM21Dr	ACGTATCTAGATCTTGCGTCATACATCGACCTG
NMPARUf	ATCTTTCTAGATTCCCGTTTCATCGGCAATC
NMPARUr	AACAAGAATTCGCGAATCGCACGCAAGAAC
NMPARDf	CCTACAAGCTTGCTTCAAGGCCTGAAACGAAC
NMPARDr	CTTGATCTAGACGCCACAGAGCCTTCCTGTC

Figure legends

Fig. S1 Growth of *P. fluorescens* Pf0-1 wild-type and mutant in PNS containing glucose (5 g/L). Vertical bars represent the standard errors of measurements done at least two independent experiments conducted in triplicate.

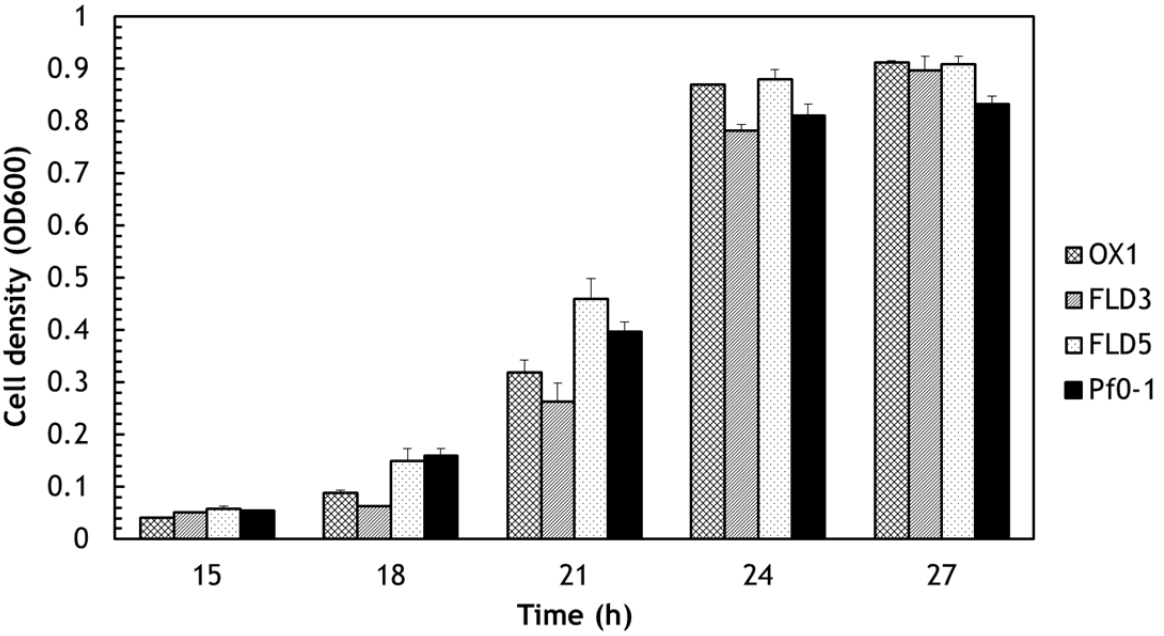
Cell density (OD600)
0
0.1
0.2
0.3
0.4
0.5
0.6
0.7
0.8
0.9
1
15
18
21
24
27
Time (h)
OX1
FLD3
FLD5
Pf0-1

Fig. S1
